# The mediating role of self-esteem in the association between perceived social support and youth mental health difficulties

**DOI:** 10.1186/s13034-026-01141-1

**Published:** 2026-07-24

**Authors:** Tamás Földi, Eszter Wittmann, Olney Rodrigues de Oliveira, Krisztina Kapornai, Enikő Kiss

**Affiliations:** 1 Department of Pediatrics and Pediatric Health Center, Department of Child and Adolescent Psychiatry, University of Szeged, Korányi fasor 14-15, Szeged, 6720 Hungary; 2 Universidade Aberta do Brasil, Fortaleza, Brazil; 3 Universidade Estadual do Ceará, Fortaleza, Brazil

**Keywords:** Child and adolescent psychiatry, Self-esteem, Social support, Mediation analysis, Psychometric profiles

## Abstract

**Background:**

This study examined whether self-esteem mediates the association between perceived social support and youth mental health difficulties in a heterogeneous sample of school-recruited youths and clinically referred inpatient and outpatient youths.

**Methods:**

The study included 312 children and adolescents (mean age = 14.36 years, SD = 2.19, range = 9–19; 37.5% boys, 62.5% girls). The primary analysis was a covariate-adjusted mediation model in the full sample, with perceived social support (MSPSS) as the predictor, self-esteem (RSE) as the mediator, and Strengths and Difficulties Questionnaire (SDQ) Total Difficulties as the outcome. Hierarchical clustering based on SDQ, RSE, and MSPSS item profiles was conducted as a secondary, exploratory analysis.

**Results:**

Higher perceived social support was associated with higher self-esteem (B = 0.26, 95% CI [0.20, 0.31], bootstrapped *p* < 0.001), and higher self-esteem was associated with fewer mental health difficulties (B = −0.43, 95% CI [−0.50, −0.35], bootstrapped *p* < 0.001) in the covariate-adjusted full-sample model. The indirect effect via self-esteem was statistically detectable (B = −0.11, 95% CI [−0.14, −0.08], bootstrapped *p* < 0.001), indicating partial mediation. Exploratory cluster-specific analyses indicated that the indirect effect was significant in the lower-difficulties/higher-resources cluster (B = −0.05, 95% CI [−0.09, −0.02], bootstrapped *p* = 0.003), but not in the higher-difficulties/lower-resources cluster (B = −0.01, 95% CI [−0.04, 0.00], bootstrapped *p* = 0.077).

**Conclusion:**

Self-esteem appears to represent one pathway linking perceived social support to fewer mental health difficulties in youth. Cluster-specific findings should be interpreted as exploratory and suggest that the strength of this pathway may vary across psychosocial profiles.

**Supplementary Information:**

The online version contains supplementary material available at https://doi.org/10.1186/s13034-026-01141-1.

## Introduction

Mental health difficulties emerging during childhood and adolescence may shape developmental trajectories with long-term consequences for psychosocial functioning and well-being [1]. Perceived social support and self-esteem are two factors that have repeatedly been linked to better mental health outcomes in young people, yet the way these factors are connected remains less clearly specified [2].

Perceived social support refers to the subjective sense that support is available from family, friends, and significant others [3]. There is a distinction between objective and subjective support, although the terms can overlap. Research in mental health usually investigates perceived support, which is subjectively experienced by the individual. Across developmental and clinical studies, perceived support is generally associated with fewer emotional and behavioral problems [4, 5].

Self-esteem, in turn, reflects a person’s global evaluation of self-worth and has consistently been associated with lower levels of depression, anxiety, and broader psychological distress [6].

A central unresolved question is whether self-esteem may partly explain how perceived social support relates to mental health. We modelled perceived social support as the predictor and self-esteem as the mediator for theory-based reasons: perceived social support may be viewed as an interpersonal resource external to the individual, whereas self-esteem reflects an internal self-evaluative process related to global self-worth [7]. This rationale is broadly consistent with the review by [3], which found that positive social support was linked to lower levels of depression, anxiety, stress, and behavioural problems, as well as to higher self-esteem and self-efficacy. In this framework, supportive relationships may foster more positive self-evaluations [8] and a greater sense of security, which in turn may be associated with fewer emotional and behavioural difficulties. At the same time, this directional specification should be understood as theory-informed rather than causal, because the present data are cross-sectional and perceived social support and self-esteem may influence each other bidirectionally [8, 9].

Beyond full-sample associations, young people may differ in their overall psychosocial profiles. A person-centered clustering approach can therefore be useful for describing psychological heterogeneity in combinations of symptoms, self-esteem, and perceived support. However, when clusters are created from the same variables later entered into mediation models, subgroup results require cautious interpretation due to reduced variance and circularity, which may influence the apparent pattern of effects.

In addition to global difficulties, domain-specific patterns also matter. Perceived social support is most consistently tied to fewer internalizing (emotional) symptoms in adolescent samples, with several studies indicating that this association is at least partly transmitted via higher self-esteem [10]. By contrast, links with externalizing domains (conduct, hyperactivity/inattention) are more heterogeneous; adolescents with elevated levels of hyperactivity and inattention symptoms frequently report lower self-esteem, but mediation of social support effects through self-esteem is less well established in these domains [11, 12]. Finally, higher prosocial tendencies are associated with greater self-worth and can be strengthened by supportive classroom climates; recognition of prosocial behaviour predicts lower subsequent depressive symptoms [13, 14]. More broadly, positive self-esteem has been linked to better psychological outcomes in adolescents, and both social support and self-esteem may function as protective factors that help maintain psychological balance and resilience in the face of mental health difficulties [15, 16]. These considerations motivate symptom group-level tests of the mediation model alongside analyses of total difficulties.

The present study had two aims. The primary aim was to test whether self-esteem mediates the association between perceived social support and mental health difficulties in the full sample, after adjustment for age, sex, and recruitment source (school vs. clinical). As a secondary exploratory extension, we used hierarchical clustering to characterize heterogeneity in overall psychosocial profiles and to examine whether this pattern appeared more or less detectable across the resulting profile groups. We did not treat clustering as an alternative to the primary mediation model, but rather as a person-centered descriptive approach to exploring heterogeneity.

We expected that greater perceived social support would be associated with fewer mental health difficulties partly through higher self-esteem. We examined possible profile-specific differences in this indirect pathway only as an exploratory secondary question and therefore did not formulate a confirmatory directional hypothesis regarding which profile would show a larger indirect effect.

## Methods

### Participants

The study was based on an original sample of 397 children and adolescents. The school group was enrolled from elementary, vocational, and high schools in Szeged, Hungary, whereas the clinical group comprised inpatients and outpatients of the Child and Adolescent Psychiatric Unit of the University of Szeged. No diagnosis-based restrictions were applied in the clinical group; youths with any psychiatric diagnosis were eligible, provided that they met the general inclusion criteria. Thus, the clinical sample was intended to reflect the diagnostically heterogeneous population of the unit rather than a disorder-specific subgroup. Inclusion criteria for both groups were being enrolled in fifth grade or above and having normal intelligence. Children below fifth grade or those who had already completed high school were not included. Participation further required parental/legal guardian consent and youth assent/consent. For the present analyses, participants with incomplete Multidimensional Scale of Perceived Social Support (MSPSS), Rosenberg Self-Esteem Scale (RSE), or Strengths and Difficulties Questionnaire (SDQ) data were excluded, resulting in a questionnaire-complete analytic sample of 312 youths (mean age = 14.36 years, SD = 2.19; range = 9–19 years).

### Procedure

After approval had been obtained from school principals, parents and children in the school group were informed about the study in writing. Consent was provided by completing the consent form and by providing parental and/or child email addresses. The questionnaire link was then sent by email to those who agreed to participate. The online survey was administered using LimeSurvey, and data were stored on a protected server of the University of Szeged, Hungary. No personal information was collected in the survey. Psychiatric participants received information and consented in person, and completed questionnaires in paper-and-pencil format, whereas school participants completed them online because of COVID-19 restrictions. Questionnaire completion in the school group was supervised by the main researcher, while data collection in the clinical group was overseen by members of the research team. Only youth self-report questionnaire data were used in the present analyses. The study was approved by the Institutional Review Board of the University of Szeged, Hungary (No. 17/2019).

### Measures

The MSPSS assesses perceived support from family, friends, and significant others [17]. The RSE measures global self-esteem [7]. The SDQ captures psychological and behavioral difficulties and prosocial strengths [18]. In line with conventional SDQ scoring, reverse-keyed items were rescored according to the manual, and the primary outcome was the 20-item Total Difficulties score, which excludes the Prosocial scale. The self-report version of the SDQ is primarily recommended for respondents aged approximately 11–17 years, depending on literacy and comprehension. In the present sample, the vast majority of participants fell within this age range; however, approximately 1% of the sample were aged 9–10 years. These cases were retained in the analyses due to their negligible proportion and because prior research suggests that SDQ self-reports from children aged 9–10 years are broadly comparable to those obtained from 11-year-olds [19]. Prosocial behavior was examined separately in exploratory subscale analyses. Internal consistencies in the present sample were excellent for MSPSS (alpha = 0.91), good for RSE (alpha = 0.86), acceptable for SDQ Total Difficulties (alpha = 0.78), and lower for the Prosocial scale (alpha = 0.64). Full descriptions are provided in the Supplementary Information.

### Statistical analysis

Analyses were conducted in R (version 4.4.2). The primary analysis was a covariate-adjusted mediation model in the full sample, with perceived social support (MSPSS total) as the predictor, self-esteem (RSE total) as the mediator, and SDQ Total Difficulties as the outcome. Age, sex, and recruitment source (school vs. clinical) were included as covariates. Path coefficients and indirect effects were estimated using nonparametric bootstrapping with 5,000 resamples. Percentile 95% confidence intervals and empirical bootstrap p-values were derived from the bootstrap distribution. For the mediation models, interpretation focused primarily on the effect estimates and bootstrap confidence intervals, while bootstrap p-values are also reported for completeness. As a secondary, exploratory analysis, hierarchical clustering (Ward’s method with Euclidean distance) was applied to min–max normalized SDQ, RSE, and MSPSS item profiles [20]. This clustering step was used as an exploratory descriptive follow-up rather than as a pre-specified confirmatory analysis. Between-cluster differences in age and questionnaire total scores were examined using Wilcoxon rank-sum tests, whereas differences in categorical sample characteristics, including sex, recruitment source, presence of any diagnosis, and comorbidity, were examined using chi-square tests. The resulting clusters were labeled post hoc according to their overall psychosocial profiles: Cluster 1 was termed the higher-difficulties/lower-resources profile, whereas Cluster 2 was termed the lower-difficulties/higher-resources profile. We then repeated the same covariate-adjusted mediation model within each cluster, retaining age, sex, and recruitment source as covariates, and conducted exploratory adjusted subscale-level indirect-effect analyses for emotional symptoms, conduct problems, hyperactivity/inattention, peer problems, and prosocial behavior. For these exploratory subscale analyses, empirical bootstrap p-values were additionally adjusted for multiple testing using the Benjamini–Hochberg false discovery rate (FDR) procedure [21] within each sample-specific subscale family. Cluster quality was summarized with the average silhouette width, agglomerative coefficient, and cophenetic correlation.

## Results

### Sample characteristics

The questionnaire-complete analytic sample comprised 312 youths. Full-sample characteristics are summarized in Table 1. The mean age was 14.36 years (SD = 2.19; range = 9–19 years), 117 participants were boys (37.5%), and 195 were girls (62.5%).

**Table 1:** Full-sample and cluster-specific sample characteristics

Characteristic	Full sample	Cluster 1	Cluster 2	*p* (Cluster 1 vs 2)
N	312	108	204	
Age, mean (SD)	14.36 (2.19)	14.68 (1.99)	14.19 (2.27)	0.068
Age range	9.0–19.0	11.0–18.6	9.0–19.0	
Boys, n (%)	117 (37.5%)	29 (26.9%)	88 (43.1%)	0.007
Girls, n (%)	195 (62.5%)	79 (73.1%)	116 (56.9%)	
School sample, n (%)	197 (63.1%)	34 (31.5%)	163 (79.9%)	< 0.001
Clinical sample, n (%)	115 (36.9%)	74 (68.5%)	41 (20.1%)	
Clinical subsample—outpatient, n (%)	35 (30.4%)	19 (25.7%)	16 (39.0%)	0.145
Clinical subsample—inpatient, n (%)	80 (69.6%)	55 (74.3%)	25 (61.0%)	
Any diagnosis, n (%)	113 (36.2%)	72 (66.7%)	41 (20.1%)	< 0.001
Comorbidity, n (%)	69 (22.1%)	51 (47.2%)	18 (8.8%)	< 0.001

Characteristics are shown for the full sample and for the two exploratory psychosocial profiles. Cluster 1 represents the higher-difficulties/lower-resources profile, whereas Cluster 2 represents the lower-difficulties/higher-resources profile. *P*-values refer to comparisons between Cluster 1 and Cluster 2. Percentages are based on the total number of participants in each column, except for outpatient/inpatient rows, where percentages are based on the clinical subsample within that column

### Full-sample mediation analysis

**Fig. 1:**
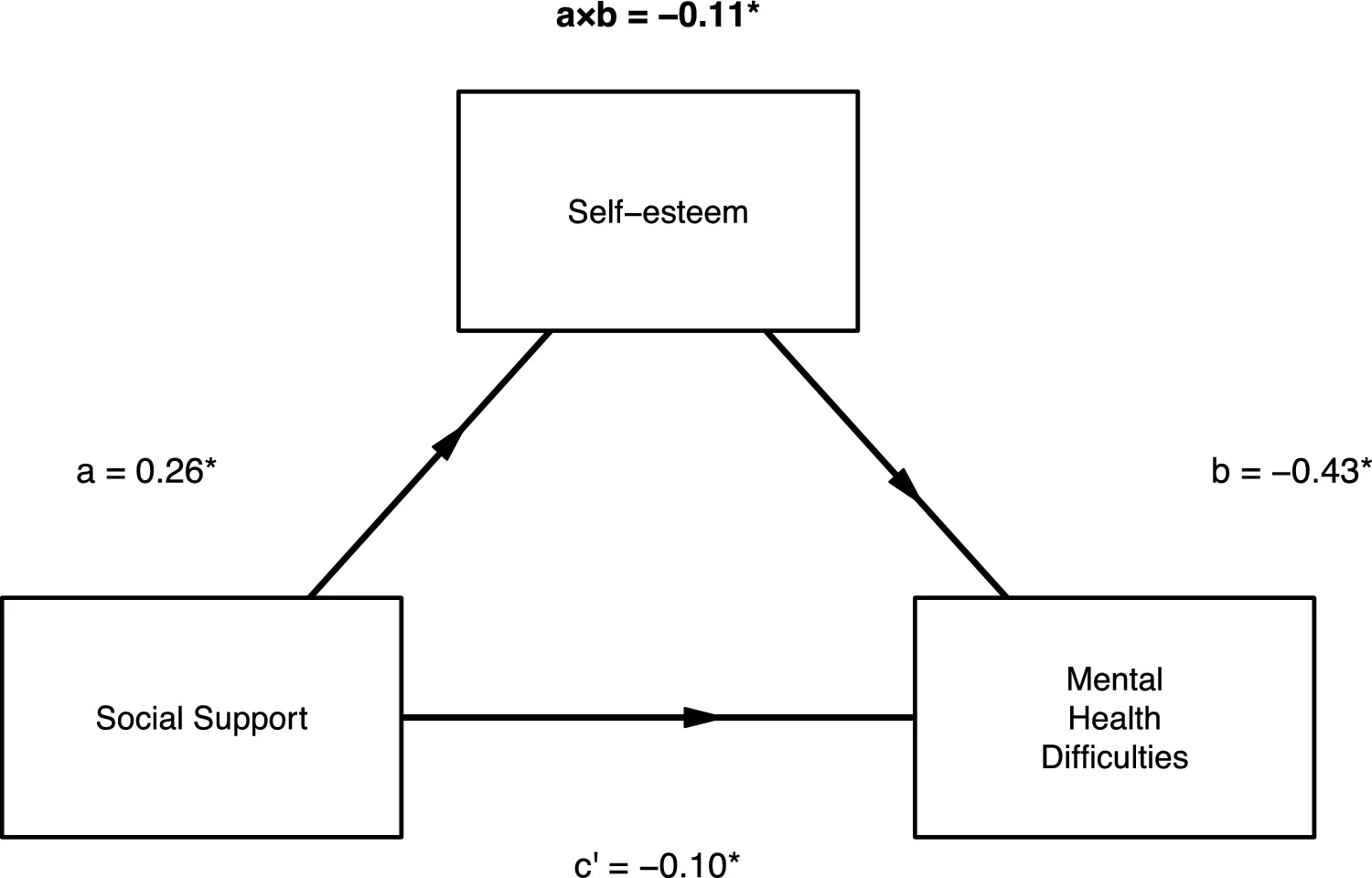
Covariate-adjusted mediation model in the full sample. Perceived social support (MSPSS total) was specified as the predictor, self-esteem (RSE total) as the mediator, and SDQ Total Difficulties as the outcome. The model was adjusted for age, sex, and recruitment source. Path coefficients are displayed together with significance markers. Asterisks indicate that the percentile 95% bootstrap confidence interval excludes zero

The covariate-adjusted mediation results for the full sample are presented in Table 2, and the corresponding model is illustrated in Fig. 1. Greater perceived social support was associated with higher self-esteem (a path: B = 0.26, 95% CI [0.20, 0.31], bootstrapped *p* < 0.001), and higher self-esteem was associated with fewer mental health difficulties (b path: B = −0.43, 95% CI [−0.50, −0.35], bootstrapped *p* < 0.001). The direct effect of perceived social support on SDQ Total Difficulties remained statistically detectable (c′ path: B = −0.10, 95% CI [−0.14, −0.06], bootstrapped *p* < 0.001). The indirect effect through self-esteem was also statistically detectable (a × b: B = −0.11, 95% CI [−0.14, −0.08], bootstrapped *p* < 0.001), indicating partial mediation.

**Table 2:** Covariate-adjusted mediation models predicting SDQ Total Difficulties in the full sample

Path	Estimate	CI lower	CI upper	Bootstrap p label	Standardized
a	0.2552	0.2002	0.308	0.0004	0.4572
b	− 0.4296	− 0.5046	− 0.3527	0.0004	− 0.5101
c′	− 0.1029	− 0.1433	− 0.0629	0.0004	− 0.219
a × b	− 0.1096	− 0.1431	− 0.0797	0.0004	− 0.2332
total	− 0.2126	− 0.2548	− 0.1729	0.0004	− 0.4522

Perceived social support (MSPSS total) was specified as the predictor, self-esteem (RSE total) as the mediator, and SDQ Total Difficulties as the outcome. All models were adjusted for age, sex, and recruitment source. Path coefficients, percentile 95% bootstrap confidence intervals, and empirical bootstrap *p*-values were estimated using 5,000 bootstrap resamples. Path labels are defined as follows: a = MSPSS → RSE, b = RSE → SDQ, c′ = direct MSPSS → SDQ effect, a × b   = indirect effect, and total = total effect

In exploratory subscale analyses conducted in the full sample, indirect effects were statistically detectable for emotional symptoms (B = −0.05, 95% CI [−0.07, −0.04], bootstrapped *p* < 0.001), conduct problems (B = −0.01, 95% CI [−0.02, −0.003], bootstrapped *p* = 0.005), hyperactivity/inattention (B = −0.03, 95% CI [−0.04, −0.02], bootstrapped *p* < 0.001), and peer problems (B = −0.02, 95% CI [−0.03, −0.01], bootstrapped *p* < 0.001), but not for prosocial behavior (B = 0.00, 95% CI [−0.004, 0.01], bootstrapped *p* = 0.293). Full-sample subscale-level indirect effects are reported in Supplementary Table 1.

### Exploratory cluster analysis and cluster-specific results

**Fig. 2:**
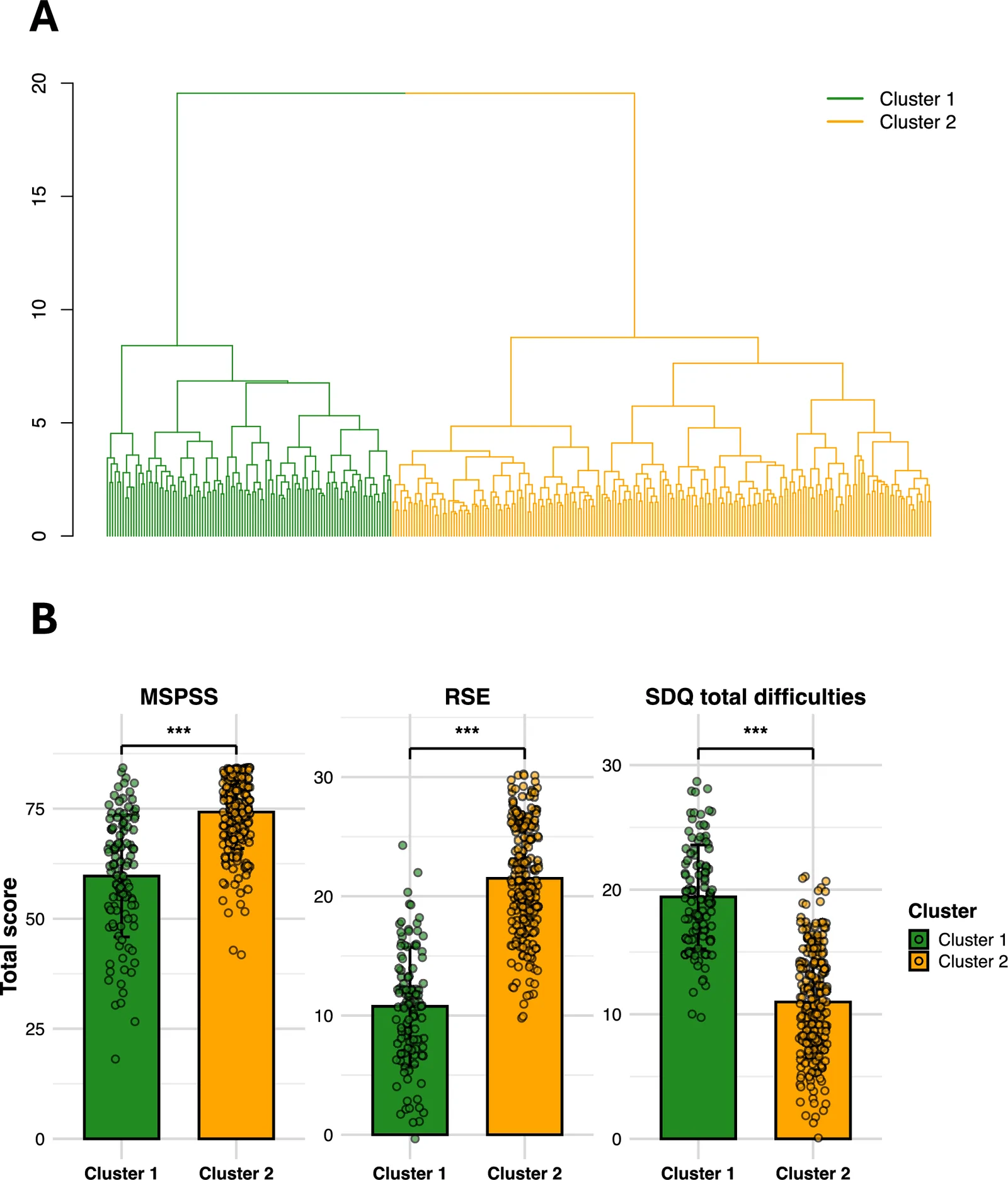
Cluster analysis results. **A** Hierarchical clustering dendrogram based on SDQ, RSE, and MSPSS item profiles. **B** Cluster differences in MSPSS, RSE, and SDQ Total Difficulties scores. Bars represent means, error bars indicate ± 1 SD, and points represent individual participants. Asterisks indicate the significance of between-cluster differences based on Wilcoxon rank-sum tests. ***p < 0.001

As a secondary, exploratory analysis, hierarchical clustering of the questionnaire-complete sample (N = 312) identified two psychosocial profile groups: Cluster 1 (n = 108), characterized by higher difficulties and lower psychosocial resources, and Cluster 2 (n = 204), characterized by lower difficulties and higher psychosocial resources. The clustering results are shown in Fig. 2, and the demographic and clinical cluster characteristics are summarized in Table 1, with detailed ICD-10 primary diagnosis frequencies by cluster reported in Supplementary Table 2.

Relative to Cluster 2, Cluster 1 showed substantially higher SDQ Total Difficulties (Mean = 19.42, SD = 4.17 vs. Mean = 10.98, SD = 4.45; *p* < 0.001), lower self-esteem (RSE: Mean = 10.77, SD = 4.95 vs. Mean = 21.51, SD = 4.77; *p* < 0.001), and lower perceived social support (MSPSS: Mean = 59.69, SD = 13.83 vs. Mean = 74.24, SD = 8.29; *p* < 0.001). Cluster 1 contained more clinically referred youths and more diagnosed/comorbid cases, whereas Cluster 2 contained more school-recruited youths (all p-values for source, diagnosis, and comorbidity < 0.001). Sex also differed between clusters (*p* = 0.007), whereas age did not (*p* = 0.068). Cluster-quality indices suggested a modest but interpretable two-cluster partition (average silhouette width = 0.17; agglomerative coefficient = 0.91; cophenetic correlation = 0.55).

In the exploratory cluster-specific mediation models, the indirect effect was not statistically detectable in Cluster 1 (B = −0.01, 95% CI [−0.04, 0.00], bootstrapped *p* = 0.077), whereas it was statistically detectable in Cluster 2 (B = −0.05, 95% CI [−0.09, −0.02], bootstrapped *p* = 0.003). Detailed cluster-specific adjusted mediation results are presented in Supplementary Fig. 1 and Supplementary Table 3.

**Fig. 3:**
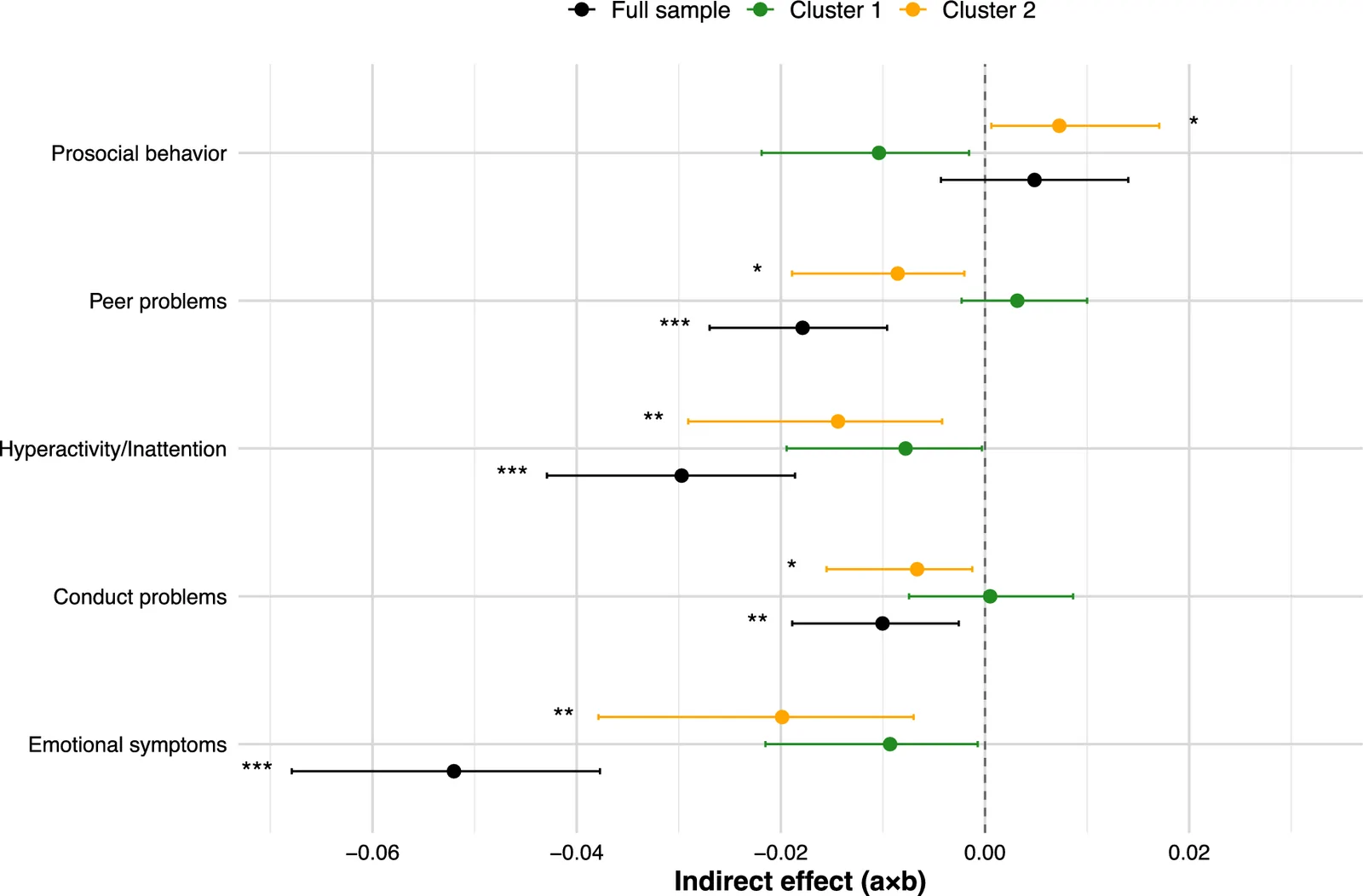
Covariate-adjusted indirect effects for SDQ subscales in the full sample and within clusters. Indirect effects (a × b) are shown for emotional symptoms, conduct problems, hyperactivity/inattention, peer problems, and prosocial behavior. Error bars represent percentile 95% bootstrap confidence intervals. Asterisks indicate Benjamini–Hochberg FDR-corrected empirical bootstrap *p*-values: *p < 0.05, **p < 0.01, ***p < 0.001. Asterisks reflect FDR-corrected *p*-values and therefore may not correspond directly to unadjusted bootstrap confidence intervals

At the cluster level, exploratory subscale analyses showed that none of the Cluster 1 indirect effects remained statistically detectable after FDR correction, whereas all five indirect effects remained statistically detectable in Cluster 2. Subscale-level indirect effects across the full sample and clusters are reported in Supplementary Table 1 and illustrated in Fig. 3.

## Discussion

The present study examined whether self-esteem mediates the association between perceived social support and youth mental health difficulties in a heterogeneous sample spanning both school-recruited and clinically referred youths. The primary finding was that, in the full sample and after adjustment for age, sex, and recruitment source, higher perceived social support was associated with fewer mental health difficulties partly through higher self-esteem. This pattern is consistent with the view that self-esteem may represent one pathway linking supportive social environments to better mental health in youth [10, 22]. By including both school-recruited and clinically referred youths, the present study may help address a gap in a literature that has often relied on higher-functioning samples.

The full-sample subscale analyses suggested that the indirect pathway through self-esteem was most evident for emotional symptoms, and to a lesser extent for hyperactivity/inattention, peer problems, and conduct problems. This pattern is broadly compatible with the idea that self-evaluative processes may be especially relevant for distress-related symptom domains [6, 10]. At the same time, the more modest findings for hyperactivity/inattention and conduct problems are in line with previous work suggesting that the role of self-esteem may be less uniform across externalizing-related domains [11, 12].

The cluster-specific findings are best understood in relation to two broad psychosocial profiles: a higher-difficulties/lower-resources profile (Cluster 1) and a lower-difficulties/higher-resources profile (Cluster 2). At the exploratory cluster level, the subscale pattern was more heterogeneous. After FDR correction, none of the Cluster 1 subscale indirect effects remained statistically detectable, whereas statistically detectable indirect effects were observed across all five subscales in Cluster 2. However, these subgroup findings should be interpreted cautiously. The clusters were derived from the same psychosocial variables that were later examined in the mediation models, therefore apparent between-cluster differences may partly reflect statistical properties of the clustering procedure, including reduced within-cluster variance and range restriction, rather than fully distinct mechanisms.

This interpretation is consistent with previous work suggesting that perceived social support and self-esteem are broadly protective, but that their associations with mental health may vary across symptom domains and levels of clinical complexity [3, 16, 23, 24]. In the lower-difficulties/higher-resources profile, the indirect pathway through self-esteem was more consistently detectable, which is in line with studies suggesting that supportive relationships may be linked to fewer mental health difficulties partly through more positive self-evaluations [10, 22]. By contrast, in the higher-difficulties/lower-resources profile, weaker or less consistent indirect effects may reflect reduced within-cluster variability, range restriction, and greater diagnostic and clinical heterogeneity. This is also compatible with the broader literature indicating that self-esteem and social support are relevant but non-specific protective factors, whose effects may be less uniform in youths with more complex or severe clinical presentations [3, 12, 15, 23]. In more symptomatic youths, self-esteem may therefore remain clinically relevant, but it is likely to operate alongside additional disorder-specific, family-level, neurodevelopmental, and symptom-focused mechanisms.

Clinically, these findings suggest that self-esteem may be one relevant pathway through which supportive relationships are associated with better mental health in youth. From a prevention and intervention perspective, this is important because both perceived support and self-esteem are potentially modifiable targets. Social support can be mobilized through family-, peer-, school-, mentoring-, and group-based approaches, and systematic review evidence suggests that such approaches may contribute to improved child and adolescent mental health when they enhance supportive relationships and connectedness [3, 25]. These implications are also consistent with classic stress-buffering and main-effect models of social support: supportive relationships may reduce the impact of stressful experiences, while also promoting well-being more generally, independent of current stress exposure [3]. In the higher-difficulties/lower-resources profile, this may be particularly relevant as part of a broader clinical approach, because strengthening family, peer, and school-based support may provide a relational foundation for more direct symptom-focused treatment. In the lower-difficulties/higher-resources profile, combined efforts to maintain supportive relationships and strengthen positive self-evaluations may have preventive value by reinforcing existing psychosocial resources. School-based social and emotional learning programmes may also improve social-emotional skills, attitudes, behaviour, and academic outcomes, providing a relevant preventive context for strengthening interpersonal resources [26]. Self-esteem-focused interventions have similarly shown beneficial effects in children and adolescents, particularly when self-esteem or self-concept is an explicit intervention target [27, 28]. In clinical settings, self-esteem may be addressed through psychoeducation, cognitive-behavioural techniques, structured group interventions, and broader mental health promotion approaches [23, 29]. However, the weaker exploratory cluster-specific findings in the higher-difficulties/lower-resources group suggest that self-esteem-focused work alone is unlikely to be sufficient for more symptomatic youths. For these individuals, interventions may need to combine relationship-based support, self-evaluative work, and more direct symptom-focused clinical treatment.

### Limitations

This study has several limitations. First, the data were cross-sectional, and therefore, the mediation models cannot establish temporal ordering or causality. The tested direction from perceived social support to self-esteem to mental health difficulties was theory-informed rather than causal. Second, the cluster-specific mediation analyses were exploratory, were not intended as pre-specified confirmatory tests, and were vulnerable to circularity because the clusters were derived from the same variables later entered into the mediation models. Finally, the prosocial scale showed lower internal consistency than the other measures, which also argues for caution in interpreting prosocial findings.

## Conclusion

In summary, self-esteem appears to be one pathway linking perceived social support to fewer mental health difficulties in youths from school and clinical settings. In the present study, the full-sample adjusted model provides the most robust evidence for this pathway, whereas the cluster-specific results are best understood as exploratory indications that its detectability may vary across psychosocial profiles.

## Supplementary Information

Additional file 1.

Additional file 2.

Additional file 3.

Additional file 4.

Additional file 5.

## Data Availability

Data supporting the findings are available from the corresponding author upon reasonable request.

## Abbreviations

SDQ: Strengths and difficulties questionnaire
MSPSS: Multidimensional scale of perceived social support
RSE: Rosenberg self-esteem scale
CI: Confidence interval
ICD: International classification of diseases
FDR: False discovery rate
