# Supplementary material for: The mediating role of self-esteem in the association between perceived social support and youth mental health difficulties
Source: Child Adolesc Psychiatry Ment Health. 2026 Jul 24;20:146. doi: 10.1186/s13034-026-01141-1 (PMC13715225; doi:10.1186/s13034-026-01141-1)
Supplement: Supplementary file 1 [file 13034_2026_1141_MOESM2_ESM.pdf]

Supplementary Figure 1. Exploratory cluster-specific mediation diagrams in Cluster 1 and Cluster 2.

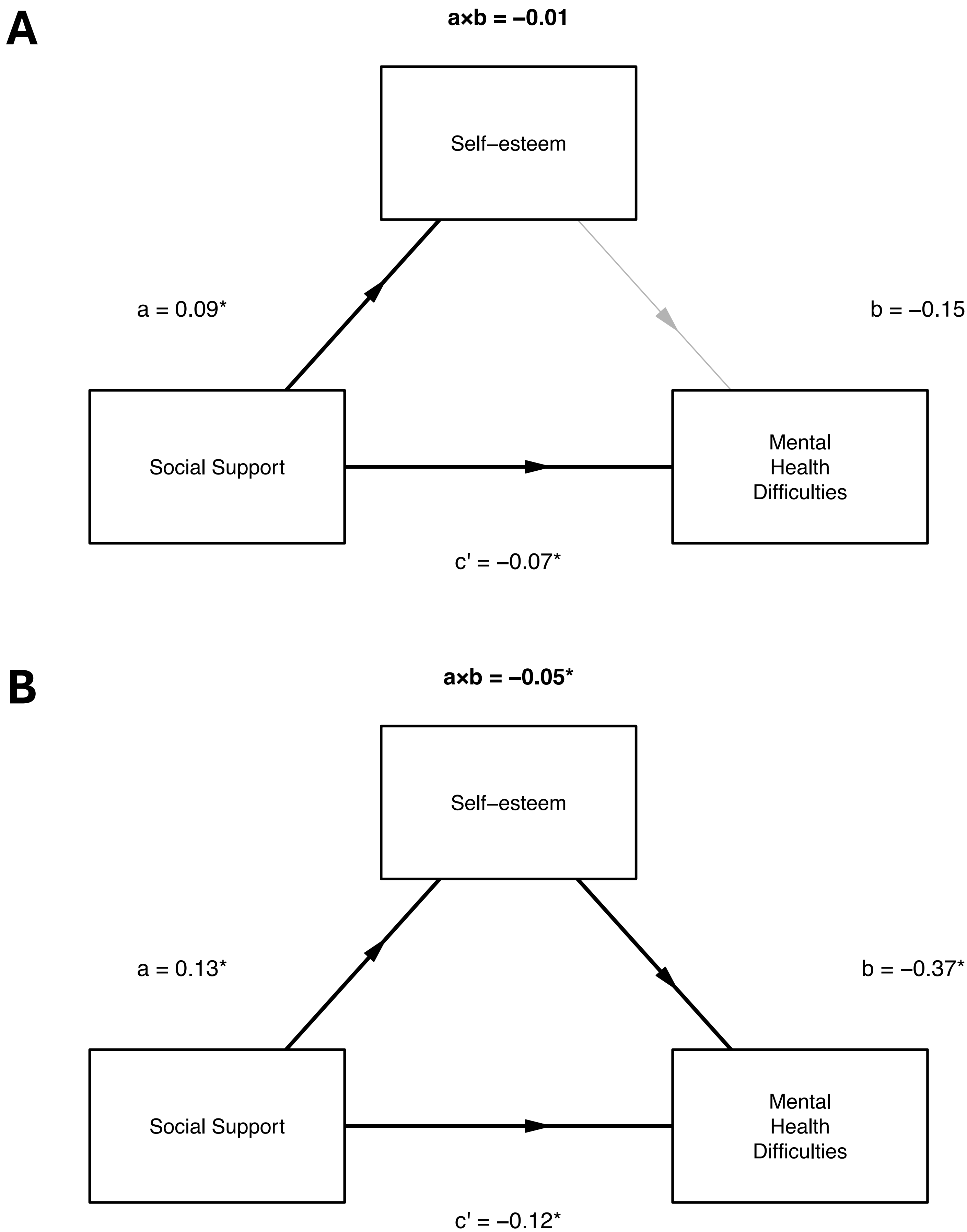

Panel A shows the mediation model in Cluster 1, representing the higher-difficulties/lower-resources profile. Panel B shows the mediation model in Cluster 2, representing the lower-difficulties/higher-resources profile. Perceived social support was specified as the predictor, self-esteem as the mediator, and SDQ Total Difficulties as the outcome. Models were adjusted for age, sex, and recruitment source. Asterisks indicate that the percentile 95% bootstrap confidence interval excludes zero. These cluster-specific analyses were exploratory secondary analyses and should be interpreted cautiously.
