# Supplementary material for: The mediating role of self-esteem in the association between perceived social support and youth mental health difficulties
Source: Child Adolesc Psychiatry Ment Health. 2026 Jul 24;20:146. doi: 10.1186/s13034-026-01141-1 (PMC13715225; doi:10.1186/s13034-026-01141-1)
Supplement: Supplementary file 2 [file 13034_2026_1141_MOESM1_ESM.docx]

Supplementary information

## Full Title

## **The Mediating Role of Self-Esteem in the Association Between Perceived Social Support and Youth Mental Health Difficulties**

## Authors

Tamás Földi^1,†^, Eszter Wittmann^1,†^, Olney Rodrigues de Oliveira^2^, Krisztina Kapornai^1^ and Enikő Kiss^1,*^

## Affiliations

^1^Department of Pediatrics and Pediatric Health Center, Department of Child and Adolescent Psychiatry, University of Szeged, Szeged, Hungary

^2^Universidade Aberta do Brasil/Universidade Estadual do Ceará, Fortaleza, Brazil

†These authors contributed equally to this work.

*Correspondence

Enikő Kiss

- Department of Pediatrics and Pediatric Health Center, Department of Child and Adolescent Psychiatry, University of Szeged, Korányi fasor 14-15, Szeged 6725, Hungary
- Tel: +36-62-342750
- E-mail: kiss.eniko.magdolna@szte.hu

## Keywords

Child and Adolescent Psychiatry, Self-Esteem, Social Support, Mediation Analysis, Psychometric Profiles

## Measures

**Multidimensional Scale of Perceived Social Support (MSPSS)**
The Multidimensional Scale of Perceived Social Support (MSPSS) is a brief, self-report questionnaire containing 12 items rated on a 7-point Likert scale ranging from 1=very strongly disagree to 7=very strongly agree. It measures how the individual perceives social support from significant others, family and friends. Higher scores represent higher degree of subjectively experienced support. MSPSS has proven to be psychometrically sound and to have good internal reliability, test-retest reliability, and robust factorial validity (Zimet et al., 1988).

**Rosenberg Self-Esteem Scale (RSE)**
Rosenberg Self-Esteem scale was developed for adolescents as a self-report scale to assess positive and negative evaluations of self (Rosenberg et al., 1989). It contains 10 items rated on a 4-point Likert scale. Respondents indicate their level of agreement on a scale ranging from 1 (strongly disagree) to 4 (strongly agree). The scores range from 0 to 30, higher scores show higher self-esteem. Original internal consistency ranged from 0.77 to 0.88, test-retest reliability from 0.82 to 0.85 (Rosenberg et al., 1989).

**Strengths and Difficulties Questionnaire (SDQ)**
SDQ is a brief behavioral screening questionnaire used for children and adolescents was developed by Goodman (1997). The scale has been validated in Hungarian youth (Turi et al., 2013). It intends to assess both psychological/behavioral problems and strengths. The prosocial scale indicates the positive prosocial characteristics, the other 4 scales (emotional symptoms, behavioral problems, hyperactivity/inattention and peer relationship problems) combined reflect total difficulties. The present study used data completed by children and adolescents about themselves.

# References

Goodman, R. (1997). The strengths and difficulties questionnaire: A research note. *Journal of Child Psychology and Psychiatry and Allied Disciplines*, *38*(5), 581–586. https://doi.org/10.1111/J.1469-7610.1997.TB01545.X

Rosenberg, M., Schooler, C., & Schoenbach, C. (1989). Self-Esteem and Adolescent Problems: Modeling Reciprocal Effects. *American Sociological Review*, *54*(6), 1004. https://doi.org/10.2307/2095720

Turi, E., Gervai, J., Áspán, N., & Halász, J. (2013). *A Képességek és Nehézségek Kérdőív (SDQ-Magy) validálása serdülőkorú klinikai populációban = Validation of the Hungarian Strengths and Difficulties Questionnaire in an adolescent clinical population*.

Zimet, G. D., Dahlem, N. W., Zimet, S. G., & Farley, G. K. (1988). The Multidimensional Scale of Perceived Social Support. *Journal of Personality Assessment*, *52*(1), 30–41. https://doi.org/10.1207/S15327752JPA5201_2

# Figure and table captions

**Supplementary Figure 1**: Exploratory cluster-specific mediation diagrams in Cluster 1 and Cluster 2.

Panel A shows the mediation model in Cluster 1, and Panel B shows the mediation model in Cluster 2. Asterisks indicate that the percentile 95% bootstrap confidence interval excludes zero. * p < .05.

**Supplementary Table 1**: Exploratory covariate-adjusted indirect effects for SDQ subscales.

Indirect effects (a×b) are shown for emotional symptoms, conduct problems, hyperactivity/inattention, peer problems, and prosocial behavior in the full sample and within Cluster 1 and Cluster 2, adjusted for age, sex, and recruitment source. Percentile 95% bootstrap confidence intervals and empirical bootstrap p-values were estimated using 5,000 bootstrap resamples. For these exploratory subscale analyses, bootstrap p-values were additionally adjusted using the Benjamini–Hochberg false discovery rate procedure. Cluster-level subscale results should be interpreted cautiously because these analyses were exploratory follow-ups to the primary full-sample model.

**Supplementary Table 2:** Distribution of ICD-10 primary diagnoses

Distribution of primary ICD-10 diagnosis groups, including participants with no recorded disorder. Percentages are based on the total number of participants within each cluster; therefore, the diagnosis categories and the no-disorder category sum to the total cluster size.

**Supplementary Table 3:** Covariate-adjusted mediation results within Cluster 1 and Cluster 2.

Perceived social support (MSPSS total) was specified as the predictor, self-esteem (RSE total) as the mediator, and SDQ Total Difficulties as the outcome. Models were adjusted for age, sex, and recruitment source and are presented as exploratory secondary analyses rather than as pre-specified confirmatory tests. Path coefficients, percentile 95% bootstrap confidence intervals, and empirical bootstrap p-values were estimated using 5,000 bootstrap resamples.
